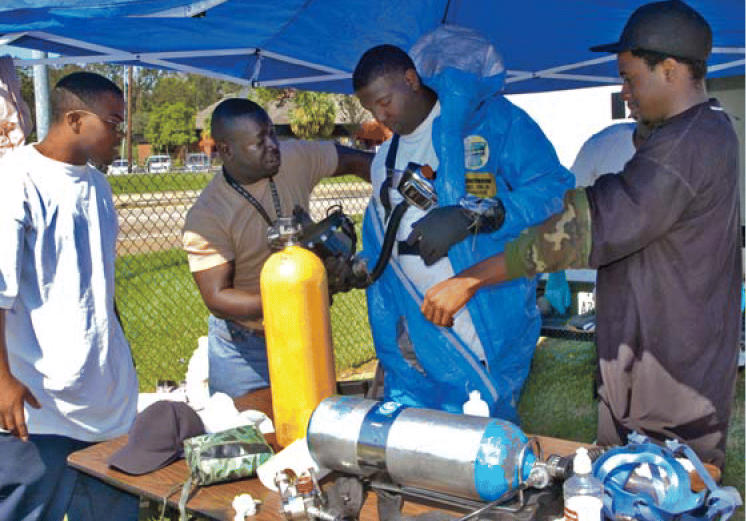# DERT WETP Katrina Response

**Published:** 2006-02

As the Gulf Coast was being overwhelmed by Hurricane Katrina on 31 August 2005, the NIEHS Worker Education and Training Program (WETP), its awardees, and its National Clearing-house for Worker Safety and Health Training developed a PowerPoint presentation that covered all of the pertinent government guidance available about safety and health hazards following the destruction caused by this hurricane. This presentation, “Protecting Yourself While Helping Others,” was posted on the NIEHS website and was updated twelve times by the end of November 2005. It was also translated into Spanish and Vietnamese by NIEHS grantees. It was then converted into a downloadable pocket guide. Thousands of copies of the Katrina safety pocket guide (http://www.wetp.org/wetp/public/hasl_get_blob.cfm?ID=2091 and http://www.wetp.org/wetp/public/hasl_get_blob.cfm?ID=2085) have been downloaded and distributed with NIEHS support. These pocket guides can accompany training sessions or can be handed out separately.

The NIEHS WETP received formal activation on 11 October by OSHA and FEMA under the Worker Safety and Health Annex of the National Response Plan. The NIEHS mission assignment is to provide safety and health training to site responders in the Katrina Recovery Zone. NIEHS WETP has collaborated with OSHA and FEMA–DHS to develop and deliver training and education programs for reducing and eliminating hazards for disaster site workers and skilled support personnel in response to Hurricane Katrina in Louisiana, Alabama, and Mississippi. NIEHS and its Worker Training Awardees will continue to develop course materials to train trainers and deliver training to target populations in the Katrina response zone including those who may serve as skilled support personnel and disaster site responders.

The focus of the current NIEHS training mission in the Gulf Coast states is to provide training to federal employees and federally deployed contractors in the four-state area of the Katrina Recovery Zone, which is comprised of Louisiana, Alabama, Mississippi, and Texas. Training activities will focus on preparing federally deployed response workers to enter highly contaminated locations and engage in site assessments, debris removal, demolition, and quality assurance activities. Safety and health training activities by NIEHS and its awardees will also be targeted to the private sector, small businesses, and local and state officials who become involved in the cleanup process.

The WETP Katrina training activities were a part of the overall NIEHS Katrina response (http://www-apps.niehs.nih.gov/katrina/).

## Contact

**James Remington** | remingtonj@niehs.nih.gov

## Figures and Tables

**Figure:**